# DNA Barcoding: Error Rates Based on Comprehensive Sampling

**DOI:** 10.1371/journal.pbio.0030422

**Published:** 2005-11-29

**Authors:** Christopher P Meyer, Gustav Paulay

**Affiliations:** **1**Florida Museum of Natural History, University of Florida, Gainesville, Florida, United States of America; Imperial College at Silwood ParkUnited Kingdom

## Abstract

DNA barcoding has attracted attention with promises to aid in species identification and discovery; however, few well-sampled datasets are available to test its performance. We provide the first examination of barcoding performance in a comprehensively sampled, diverse group (cypraeid marine gastropods, or cowries). We utilize previous methods for testing performance and employ a novel phylogenetic approach to calculate intraspecific variation and interspecific divergence. Error rates are estimated for (1) identifying samples against a well-characterized phylogeny, and (2) assisting in species discovery for partially known groups. We find that the lowest overall error for species identification is 4%. In contrast, barcoding performs poorly in incompletely sampled groups. Here, species delineation relies on the use of thresholds, set to differentiate between intraspecific variation and interspecific divergence. Whereas proponents envision a “barcoding gap” between the two, we find substantial overlap, leading to minimal error rates of ~17% in cowries. Moreover, error rates double if only traditionally recognized species are analyzed. Thus, DNA barcoding holds promise for identification in taxonomically well-understood and thoroughly sampled clades. However, the use of thresholds does not bode well for delineating closely related species in taxonomically understudied groups. The promise of barcoding will be realized only if based on solid taxonomic foundations.

## Abstract 2

An examination of the efficacy of barcoding using a comprehensive sample of marine gastropods reveals that the method performs poorly for identifying closely related species in taxonomically understudied groups.

## Introduction

### The Controversy

DNA barcoding, the recently proposed DNA-based project for species identification, has attracted much attention and controversy [1–6]. Proponents envision that a short fragment of DNA can be used to diagnose taxa, increasing the speed, objectivity, and efficiency of species identification. Initial tests of genetic barcoding using mitochondrial markers on animals reported near-100% accuracy, indicating that the method can be highly accurate under certain conditions [1,7,8]. Accurate species identification—assignment of an unknown to a known—requires a comprehensive comparative molecular database against which unknowns can be compared. However, it is clear that most of the biological diversity in the world is undocumented [9,10]. Therefore, a stated second goal of DNA barcoding is to facilitate the species-discovery process [11–13]. Such a proposal has raised the concern of the systematics community, which claims that adopting barcoding would be a step backwards [14–16], returning taxonomy to typology [17]. Opponents also note that mitochondrial DNA (mtDNA) sequences alone may be insufficient to diagnose species, because genetic differentiation does not necessarily track species boundaries [18,19]. Thus, Funk and Omland [20] found that ca. 23% of surveyed metazoan species are genetically polyphyletic or paraphyletic, implying that they would not be differentiable by barcoding techniques.

### What Does Accuracy Depend on? The Barcoding “Gap”

The critical issue in barcoding is accuracy. How well does a single gene sequence perform in delineating and identifying species? Accuracy depends especially on the extent of, and separation between, intraspecific variation and interspecific divergence in the selected marker. The more overlap there is between genetic variation within species and divergence separating sister species, the less effective barcoding becomes. Initial efforts to test barcoding suggested a significant barcoding “gap” between intra- and interspecific variation, but these efforts have greatly undersampled both intraspecific variation (mostly 1–2 individuals per species sampled) and interspecific divergence (because of incomplete or geographically restricted sampling) [1,7,8].

### Overlap between Intra- versus Interspecific Variation

**Figure 1:**
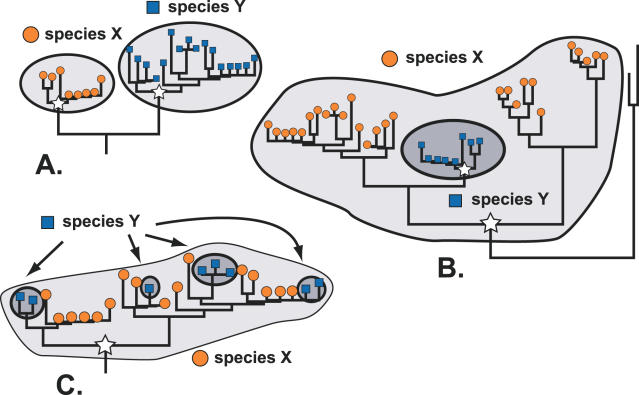
Phylogenetic Relationships and Terminology (A) Reciprocal monophyly. Members of each species share a unique common ancestor. For each species, the white star represents the coalescent, the point at which all extant haplotypes share a common ancestry. (B) Paraphyly. One species (Y), is monophyletic, but nests within another recognized species (X). Thus, the coalescent of species Y (small star) is contained within the coalescent of species X (large star). (C) Polyphyly. Neither species X or Y are monophyletic, and both coalesce to the white star.

When the coalescent has yet to sort between incipient species (ancestral polymorphism), intraspecific variation overlaps with interspecific divergence and gives rise to genetically polyphyletic or paraphyletic species (Figure 1) [18,21,22]. When such overlap is real (i.e., not the result of poor taxonomy), then that marker cannot reliably distinguish among those species. Overlap between intraspecific and interspecific variation can also occur broadly within a tree, even when each species is reciprocally monophyletic to all others. This occurs when intraspecific variation in parts of the tree exceeds interspecific divergence in other parts of the tree—i.e., when the range of intra- and interspecific variation overlaps. Such overlap will not affect identification of unknowns in a thoroughly sampled tree, where they should fall within the coalescent of already characterized species. However, such overlap can have a substantial impact during the discovery phase (i.e., in an incompletely sampled group), as the status of unknowns that fall outside the coalescent of previously sampled species is problematic to evaluate.

### Gap versus Overlap: The Efficacy of Thresholds

**Figure 2:**
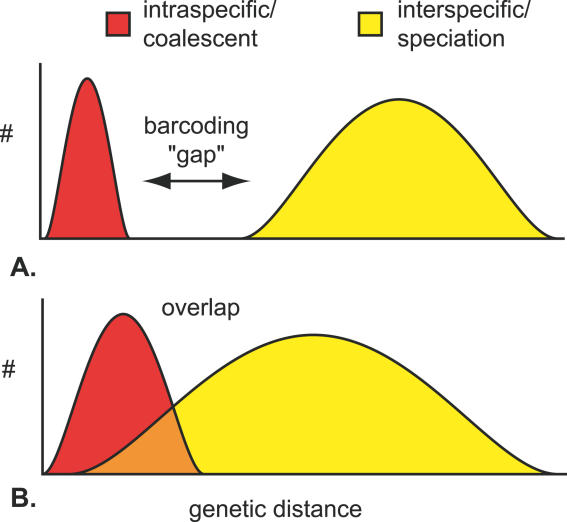
Schematic of the Inferred Barcoding Gap The distribution of intraspecific variation is shown in red, and interspecific divergence in yellow. (A) Ideal world for barcoding, with discrete distributions and no overlap. (B) An alternative version of the world with significant overlap and no gap.

The proposed mechanism for the evaluation of unknowns within a partially sampled phylogeny is through the implementation of thresholds, chosen to separate intraspecific variation from interspecific differences. An unknown differing from an existing sample by less than a threshold value is assumed to represent that species, but one differing from existing sequences by more than the threshold value is assumed to represent a new taxon. This method is vulnerable to both false positives and false negatives. False positives are the identification of spurious novel taxa (splitting) within a species whose intraspecific variation extends deeper than the threshold value; false negatives are inaccurate identification (lumping) within a cluster of taxa whose interspecific divergences are shallower than the proposed value. The accuracy of a threshold-based approach critically depends upon the level of overlap between intra- and interspecific variation across a phylogeny. While Hebert et al. [7] suggest that a wide gap between intra- and interspecific variation makes a threshold approach promising (Figure 2A), Moritz and Cicero [23] argue that the overlap is considerably greater when a larger proportion of closely related taxa are included, making the method problematic (Figure 2B). To evaluate the performance of this method, we need to assess the extent of and overlap between intra- and interspecific genetic variation comprehensively, within a thoroughly sampled clade [23,24].

Here we present the first dataset sufficiently comprehensive to robustly evaluate the efficacy of DNA barcoding: the cowrie genetic database [25,26]. This dataset includes sequences from >2,000 individuals in 263 taxa, representing >93% of recognized cowrie (marine gastropods of the family Cypraeidae) species worldwide, with multiple individuals from >80%, and at least five individuals from >50% of the taxa. These data provide near comprehensive sister-species coverage, and a broad survey of intraspecific variation. We use this dataset to address several questions. How accurate is molecular identification of unknowns in a thoroughly sampled tree? What are the reasons for failures in such identifications? How much do intraspecific variation and interspecific divergence overlap across this well-sampled phylogeny? How much error is associated with threshold-based identifications, and what threshold value minimizes this error? Finally we use data from two smaller but similarly exhaustively sampled clades (of limpets [27] and turbinid gastropods [28]), to evaluate the generality of these patterns. Cowries encompass a diversity of species attributes: recent versus ancient, planktonic versus direct development, common versus rare, and large Indo-west Pacific-wide ranges versus single island endemics. All cowries have internal fertilization, mostly with feeding larvae, whereas limpets and turbinids are external fertilizers, with non-feeding larvae. While all three examples are gastropods, their range of species attributes implies that these findings are likely applicable to a wide range of taxa.

The effectiveness of barcoding is critically dependent upon species delineation: splitting decreases while lumping increases both intraspecific variation and interspecific divergence. Taxonomically, cowries are one of the most extensively studied marine gastropod families, both morphologically [29–33] and genetically [25,26], thus their species are well circumscribed. We analyze and compare barcoding performance for two types of species-level taxa based on different levels of taxonomic analysis: (1) traditional, morphological species, as defined by the most recent morphology-based revision [33], and (2) evolutionary significant units (ESUs), as defined through an integrative taxonomic analysis of combined and extensively sampled genetic and morphological data (slightly modified from [25,26]). We thus compare the efficacy of barcoding across a 2 × 2 matrix: performance with traditional species versus ESUs in an identification versus discovery setting. Traditional species provide a test of barcoding when substantial morphological information is available, but remain untested with genetic tools. This level of knowledge is comparable to biotic checklists, which are often used to guide sampling in barcoding efforts. In contrast, ESUs provide the best of integrative taxonomy, a system where population-level and geographically extensive genetic sampling has tested species-level boundaries described by extensive morphological studies. Because ESUs are defined as reciprocally monophyletic units, they exclude the possibility of, and errors associated with, paraphyletic or polyphyletic species, and thus provide the optimal units for barcoding. Given that at present their reciprocal monophyletic status is based on the same genetic marker used for barcoding, they should lead to 100% accuracy in species identification tests. Presently, cowrie ESUs exclude potentially valid, young species that are not reciprocally monophyletic in cytochrome *c* oxidase I (COI) sequences; however, additional work may demonstrate some of these to be valid species. ESUs fulfill the phylogenetic species concept; however, we choose to recognize them only as ESUs, to emphasize that although they are genetically divergent and distinctive, they all are not, or destined to become, biological species.

The correspondence between ESU definitions and traditional morphological taxonomy is high. Remarkably, 255 ESUs (97%) have been recognized previously at either the specific or subspecific level and are therefore supported by independent morphological criteria in addition to molecular data. Only eight ESUs are genetically distinct but have not been previously recognized by traditional taxonomy; all of these are allopatric, genetically divergent lineages. So defined, the 263 ESUs sampled include >93% of the 233 recognized cowrie species and 56 recognized subspecies. From here on, we use “ESU” to denote taxa recognized through an integrated approach with the aid of molecular criteria, and “species” to refer to taxa recognized at that level in traditional cowrie taxonomy. The same definition led to the recognition of 12 ESUs in the *Patelloida profunda* group of limpets [27] and 30 ESUs in the *Astralium rhodostomum* complex of turbinid gastropods [28]. In both groups traditional taxonomic study lags substantially behind cowries, and many ESUs represent undescribed, but morphologically recognizable, species.

## Results/Discussion

### Accuracy in Thoroughly Sampled Phylogenies: Identification

Identification of unknowns against a thoroughly sampled phylogeny was prone to error when traditional species were utilized, but accurate when ESUs formed the basis of the phylogeny. Assignment of unknowns to a phylogeny comprised of exemplars of every traditional species was correct 80% of the time using a neighbor-joining approach (see Materials and Methods). Eight percent of the assignments were incorrect, while 12% were ambiguous, with the unknown falling as sister to a clade comprised of its species plus its sister species. Parsimony analyses were unambiguously correct 79%, incorrect 7%, and ambiguous 10% of the time, while the correct placement was one of multiple, equally parsimonious placements in 4% of the cases. Ambiguous assignments also represent failures of the barcoding method, as although the unknowns “belong” to sampled species, they fall outside of that species as characterized by an exemplar approach, and could represent a novel taxon. This approximately 20% failure rate at the species level is consistent with Funk and Omland's [20] assessment that 23% of metazoan species are not monophyletic.

In contrast, identification of unknowns was 98% accurate with a neighbor-joining approach against an ESU phylogeny. Similar analyses of turbinid and limpet datasets had success rates of 100% and 99%, respectively. These results are not unexpected, however, as the reciprocal monophyly criteria for circumscribing units predisposed the system for success. More surprising is the 2% failure rate (1% each from incorrect assignment and ambiguity). In these incorrect identifications, improper assignment involved a recently derived sister ESU. These failures occur because only a single exemplar was used to define ESUs in the phylogenies. The rooting of the three-taxon arrangement between the sample, correct ESU, and sister ESU is tenuous, and vulnerable to artifacts of incomplete sampling. If all sequenced haplotypes were included in the analyses, the unknown would have been correctly assigned. Nevertheless, these high success rates are encouraging, particularly since only a single exemplar was used for comparison [34], and many of the divergences between sister taxa are shallow.

**Figure 3:**
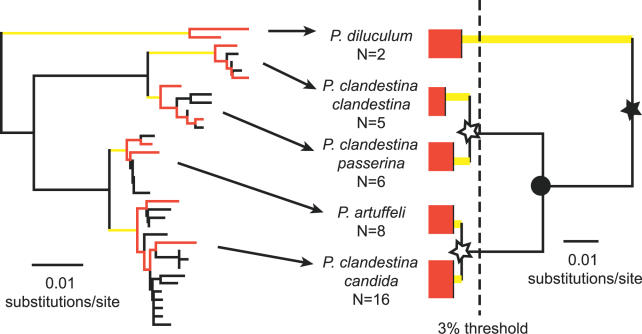
Intraspecific and Interspecific Estimations A subclade of five cowrie ESUs shows how both coalescent and divergence depths are generated. The two most disparate individuals are culled from within each ESU (left—red) and used in a constrained phylogeny with a molecular clock enforced (right) to recover both the maximum coalescent depth (red) and the divergence depths between sisters (yellow). Two young ESUs (stars) would be missed (false negatives) if a 3% threshold cutoff (shown) was employed. Note that *Palmadusta artuffeli,* a Japanese endemic species, is nested among monophyletic subspecies of the paraphyletic species *P. clandestina*. The black circle indicates the coalescent for the species *P. clandestina,* and the black star indicates the interspecific divergence for species-level analyses.

What are the sources for the 20% failure rate in species-level analyses? Non-monophyly at the species level leads to barcoding failure both in thoroughly sampled and threshold approaches, and represents the greatest challenge for the method. Funk and Omland [20] recognize five reasons for species-level non-monophyly; two of these account for most non-monophyly in cowries: imperfect taxonomy and incomplete lineage sorting. Imperfect taxonomy can cause non-monophyly either through lack of recognition of multiple taxa within a traditional species (overlumping) or when morphotypes are inappropriately recognized as species (oversplitting). Overlumping is common in cowries and readily identified via thorough genetic sampling: 16 recognized cowrie species (7%) are nested ESUs within other, paraphyletic species comprised of multiple ESUs (e.g., *Palmadusta artuffeli* within *P. clandestina*; Figure 3). Oversplitting is more difficult to resolve because young species that remain within their sister species' coalescent lead to the same polyphyletic, genetic signature. Of 218 traditional cowrie species tested [25,26], 18 (8%) are polyphyletic with respect to another recognized species. These are either young species (incomplete lineage sorting), or artificially split forms (imperfect taxonomy); additional research is needed to resolve their status. Note that such young species are also neglected by the ESU approach and represent the ultimate limit for barcoding: non-monophyly that cannot be eliminated at the marker (COI) used.

Using the ESU concept in hindsight, we can ascribe the failures in our species-level test to artifacts of paraphyly or polyphyly (Figure 1). Ten percent of the failures can be attributed to overlumped, paraphyletic species, while nine percent are the results of either oversplit or young (incompletely sorted) polyphyletic species. The remaining 1% is real error based on single exemplars of the type mentioned previously.

The other three causes of species non-monophyly (inadequate phylogenetic information, unrecognized paralogy, and introgression) identified by Funk and Omland [20] are of minor importance in these studies. Since all three gastropod datasets are well circumscribed using morphological, anatomical, geographic, and molecular attributes, we have minimized the problems of inadequate phylogenetic information. We can estimate error rates associated with paralogy and the presence of nuclear copies of mtDNA (NUMTs; [35]). In generating sequence data for 2,026 cowrie individuals, seven sequences (0.3%) have been generated that are thought to be NUMTs, all within three species. Low levels of NUMTs (<1%) were also reported by Hebert et al. [12] in their study of *Astraptes* butterflies. NUMTs can be problematic in some taxa (e.g., [36]), but their presence is usually ascertained by translation shifts in amino acid patterns or signal deterioration in electropherograms derived from non-cloned products. The final source of non-monophyly is introgression. Hybrid individuals have been reported within cowries, and indeed, mtDNA data reveal that individuals assigned conchologically to certain species or subspecies possess haplotypes of closely related lineages, indicating some past introgressive or hybridization event. Using only mtDNA sequences, these individuals would be identified incorrectly. How frequently does this occur? Less than 2% of cowrie individuals, 1% of turbinids, and 0% of limpets possess COI sequences inconsistent with their morphology, indicating that the impact of introgression has been minor. Nevertheless, this low frequency should be included in error estimation. Therefore, our overall empirical error in the best of situations (ESUs) for species identification is 4%–12%: 2% because of the use of single exemplars, 2% from introgression, and 0%–8% from polyphyletic species.

### Accuracy in Undersampled Phylogenies Using Thresholds: Discovery

To evaluate the efficacy of thresholds for species delineation in a partially sampled clade, we examined the overlap between intra- and intertaxon divergences at both ESU and species levels using a phylogenetic approach. Three different metrics were used to characterize intraspecific variation: (1) average pairwise intraspecific difference (K2P distance) between all individuals sampled within species/ESU, as employed by previous researchers [7,8]; (2) average theta (θ), where theta is the mean pairwise distance within each taxon, thereby eliminating bias associated with uneven sampling among taxa; and (3) average coalescent depth, the depth of the node linking all sampled extant members of a taxon, bookending intraspecific variability (see Materials and Methods). Genetic distance between terminal taxa and their closest sister was used to characterize interspecific divergence.

**Figure 4:**
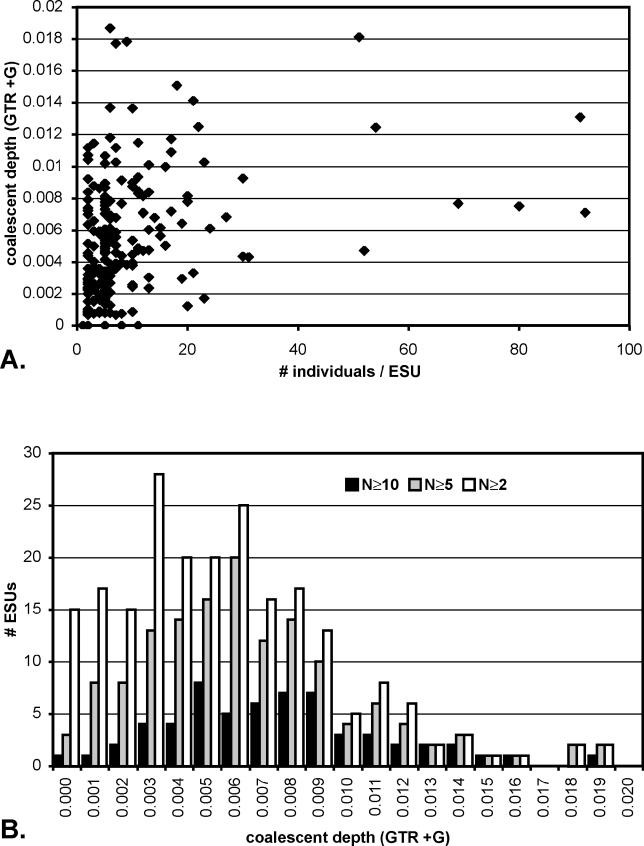
Sample Size Effect on Intraspecific Variation A. Coalescent depth vs. sample size. B. Histograms for coalescent depths of various sample size classes. Mean coalescent depth increases with increased sample size, from 0.0049 for *n* ≥ 2, to 0.0057 for *n* ≥ 5, to 0.0068 to 0.0070 for *n* ≥ 10.

**Figure 5:**
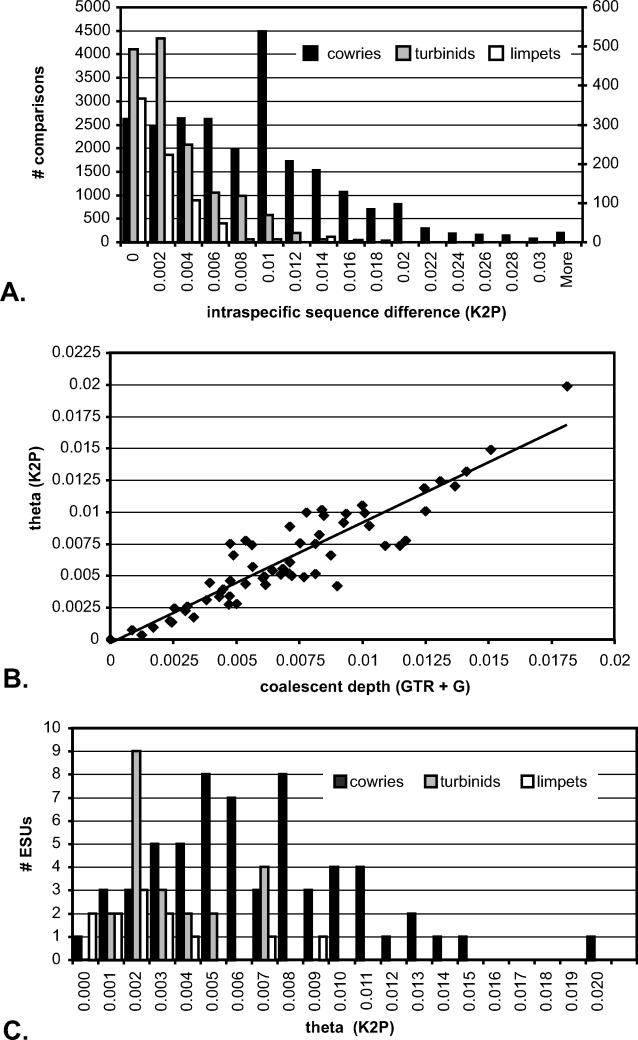
Alternative Metrics for Intraspecific Variation (A) Distribution of all intraspecific pairwise K2P distances for cowrie ESUs with *n* ≥ 10, turbinids and limpets. Left *y*-axis for cowries; right *y*-axis for others. (B) Comparison between estimated theta versus estimated maximum coalescent for each cowrie ESU with *n* ≥ 10; r^2^ = 0.837. (C) Distribution of theta values for cowrie (*n* ≥ 10), turbinid and limpet ESUs.

A wide range of intraspecific variation was encountered among ESUs in all three datasets, with generally less variation in turbinids and limpets than cowries. Sampling effort was designed to capture the greatest intraspecific variation by targeting the most disparate populations in a taxon, whenever possible. Thus, while coalescent depths generally increase with sample size (Figure 4A), they are variable, and ESUs with *n* ≥ 2, *n* ≥ 5, and *n* ≥ 10 samples overlap broadly (Figure 4B). The distribution of all intraspecific, pairwise genetic distances approximates a Poisson distribution (Figure 5A). Calculated values of theta for cowrie ESUs with ≥ten samples are normally distributed, and are highly correlated with estimated coalescent depth (Figure 5B). All three measures of intraspecific variability (average pairwise distances, theta, and coalescent depth) are substantially higher in cowries than in turbinids or limpets (Table 1). This may be a result of smaller effective population sizes in the latter two groups [37], reflecting their poor dispersal abilities because of non-feeding larvae, and resultant narrow ESU ranges. A similar pattern is evident within cowries: taxa that lack planktonic larvae and consequently have restricted dispersal and narrow ranges, have a smaller mean theta (0.0029) than cowries that possess planktonic larvae (0.0070).

**Table 1:**
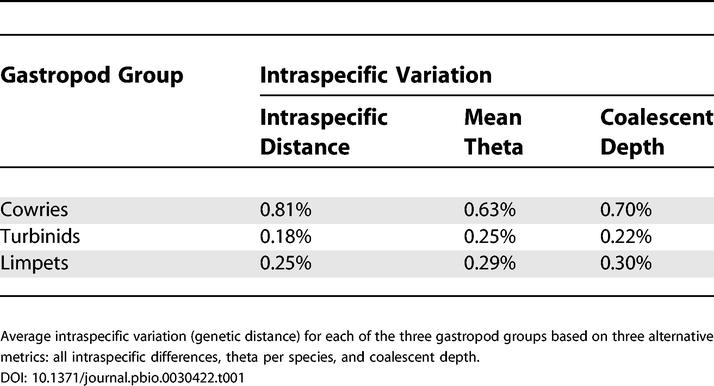
Intraspecific Variation

**Figure 6:**
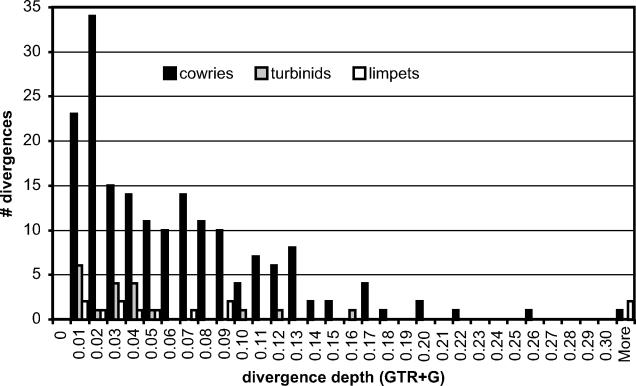
Interspecific Variation Distribution of divergence depths between terminal ESUs and their sister ESU(s) in cowries, turbinids and limpets.

As with intraspecific variation, a wide range of interspecific differences is found in all three gastropod groups, indicating that divergences are spread out over time (Figure 6). It is interesting to note that intraspecific variation (as measured by coalescent depth) is not correlated with interspecific divergence for ESUs ≥ five individuals (*p* = 0.12), indicating that older species (those without close extant relatives) do not have more intraspecific variation than younger species (those with close relatives).

#### Gap or overlap? Efficacy of thresholds with ESUs

We found broad overlap between levels of intraspecific variation and interspecific divergence at the ESU level in cowries. Intraspecific variation is well constrained: only five ESUs (2%–3% of ESUs with *n* ≥ 10, *n* ≥ 5, and *n* ≥ 2 samples/ESU) have coalescent depths >1.5% (=3% threshold), and none have >2% (=4% threshold) (Table 2). Coalescent depths are recorded as nodal depths, and thus are half the value of pairwise distances commonly reported for threshold values. Therefore, if an unknown was >3% divergent from all other samples, we could say with ~98% confidence that it represents an independent evolutionary lineage. Such false-positive errors become rapidly more common at lower thresholds, as 20%, 15%, 11% of ESUs (with *n* ≥ 10, *n* ≥ 5, and *n* ≥ 2 samples/ESU) have coalescent depths >1% (=2% threshold). In turbinids and limpets, all coalescent depths are <1%, thus none yield a false positive at even a 2% threshold. Because these error rates are determined by maximum coalescent depth, this assessment of performance is conservative. Two randomly chosen individuals within an ESU will likely be less divergent than the two most disparate individuals. For direct comparison with Hebert et al. [7] and Barrett and Hebert [8], examination of all intraspecific pairwise distances (Figure 5A) yields 99% and 95% confidence values at thresholds set at 2.85% and 1.99% in cowries, 1.12% and 0.81% for turbinids, and 1.38% and 0.52% for limpets, respectively.

**Table 2:**
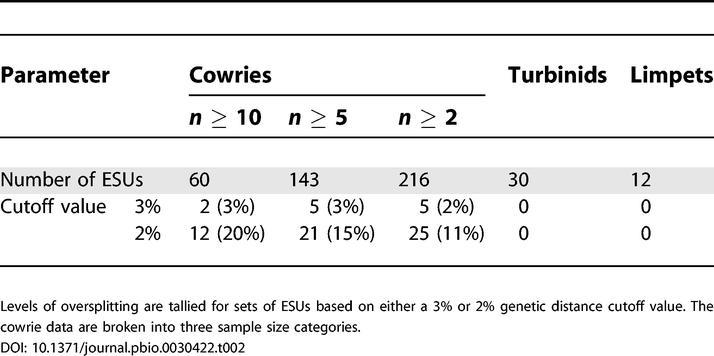
False-Positive Error Rates

**Figure 7:**
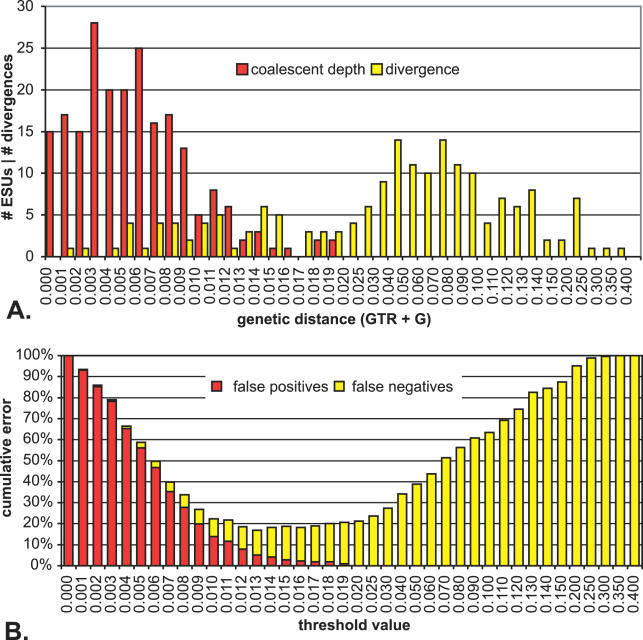
Barcoding Overlap: Cowrie ESUs (A) Relative distributions of intraspecific variability (coalescent depth—red) and interspecific divergence between ESUs (yellow), demonstrating significant overlap and the lack of a barcoding gap. Note that the *x*-axis scale shifts to progressively greater increments above 0.02. (B) Cumulative error based on false positives plus false negatives for each threshold value. The optimum threshold value is 0.013 (2.6%), where error is minimized at 17%.

In contrast, interspecific ESU divergences are much less constrained, extending at their lower end well into the range of intraspecific variation (Figure 7A). Thus, high divergence thresholds miss many young ESUs. Of the 263 cowrie ESUs sampled, 16% would be artificially lumped with another ESU at a 3% threshold, and 8% would be lumped even at a 2% cutoff (Table 3). Most (79% at the 3% threshold) of the lumped ESUs are allopatrically distributed sister taxa, yet more than half (22 of 42) are traditionally recognized species. A similar percentage of taxa would be overlooked at a 3% cutoff in turbinids (20%) and limpets (17%) (Table 3). This high incidence of false negatives reflects both the comprehensive phylogenetic sampling and increased taxonomic scrutiny these taxa have received.

**Table 3:**
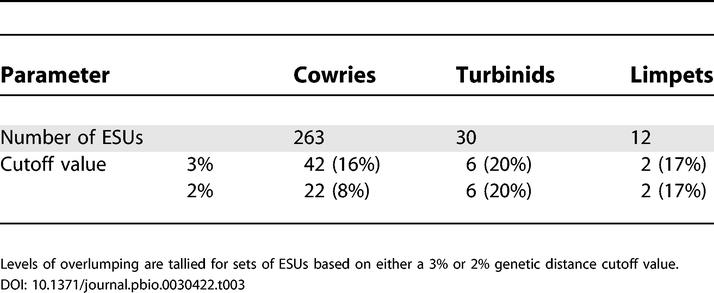
False-Negative Error Rates

How high should thresholds be set to minimize error? False-positive and false-negative error rates can be totaled for any threshold value across the phylogeny, and combined error minimized (Figure 7B). In cowries the lowest overall error (17%) was at a threshold values of 2.6%, and error varied little (17%–19%) between 2.4%–3.4% thresholds. Errors at these levels are largely the result of missing young taxa, not of false recognition of additional species. In turbinids, as in cowries, the distribution of intra- and interspecific divergences overlap, and combined error is lowest (7%) at thresholds values of 1.2%–1.6% (Figure S1). In contrast, thresholds are effective and error can be entirely eliminated in limpets: there is no overlap at a threshold of 1.7% (Figure S2). The better performance of turbinids and limpets is likely in part the result of their shallower coalescents and lower diversity.

A 3% threshold has been cited as sufficient genetic disparity to characterize different species [1]. The actual threshold value that researchers would be willing to accept as indicative of a new taxon, if any, varies depending upon philosophy, marker choice, and group of organisms. For our three datasets, a 3% threshold would work well at minimizing false positives, but it would create many false negatives. Alternatively, Hebert et al. [7], in order to screen for novel taxa, proposed to set a standard sequence threshold value that minimizes false positives at ten times the mean intraspecific variation. This would set the threshold at around 8% in cowries (based on 60 ESUs with *n* ≥ 10; Table 1), well above the 4% level where all false positives are eliminated, considerably above the optimum (2.6%), and leading to a 34% error rate (all false negatives).

Substantial variation in the relationship between intraspecific variation and optimal threshold values to either minimize combined error or to eliminate all false positives makes setting the latter on the former problematic. The optimum threshold values to minimize total error correspond to 3.2–4.1 times the level of intraspecific variation in cowries, depending on which measure is used (Table 4). The factors range from 4.9–6.3× if one were to use a conservative threshold that eliminates false positives. The corresponding ranges for turbinids are 4.8–7.8×, while for limpets it is at 5.7–6.8× (Table 4). The range of values among these gastropods and Hebert et al.'s [7] bird samples indicate that no simple formula based on intraspecific variation will yield a robust threshold to minimize error across groups.

**Table 4:**
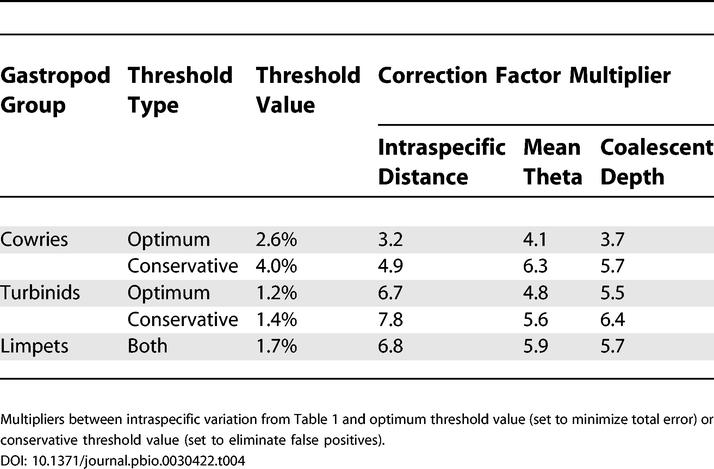
Threshold Correction Factors

Thresholds can be used to either minimize total error or to cleanly screen for novel taxa. Our results imply that they serve poorly for the former, as high error rates remain at even optimal threshold values. However thresholds can certainly be set in a way to guarantee that sequences beyond them represent novel taxa. In cowries, a 4% screening threshold eliminates all known intra-ESU variants, and guarantees that such divergent taxa are novel. However the same threshold will also miss 21% of novel taxa, as they will register less divergent. Thus, thresholds can assist in the species discovery process by guaranteeing the distinctiveness of genetically deep variants, at some cost.

#### Efficacy of thresholds with traditional taxonomy

**Figure 8:**
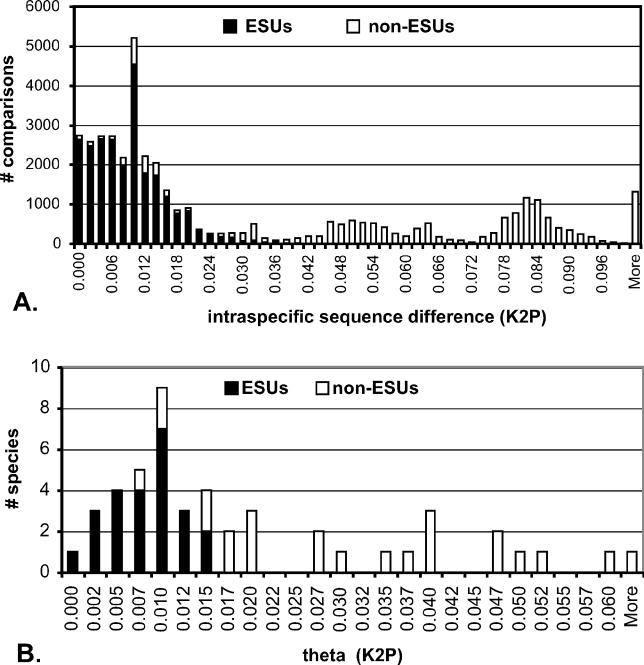
Intraspecific Variation Based on Recognized Species (A) The distribution of all intraspecific pairwise distances for traditionally recognized cowrie species with *n* ≥ 10. The white bars represent intraspecific distances where the two specimens compared fall into separate ESUs. (B) Theta values for traditionally recognized cowrie species. Black bars are species that correspond to an ESU; white bars are species that include multiple ESUs.

**Figure 9:**
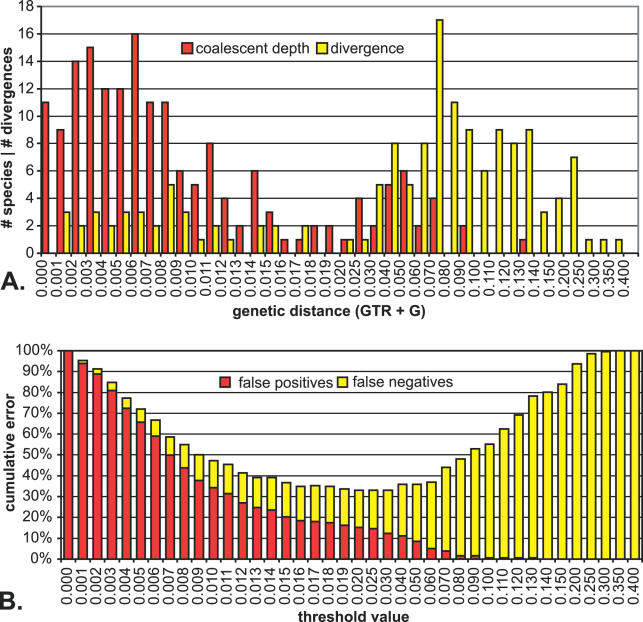
Barcoding Overlap: Cowrie Species Data are presented as in Figure 7; however, estimates of intraspecific variation and interspecific divergence are based on traditionally recognized cowrie species. (A) Relative distributions of intraspecific variability (coalescent depth—red) and interspecific divergence between species (yellow), demonstrating a more pronounced overlap than when utilizing ESUs. Note that the *x*-axis scale shifts to progressively greater increments above 0.02. (B) Cumulative error based on false positives plus false negatives for each threshold value. The optimum threshold value is 0.025 (5%), where error is minimized at 33%.

Error rates almost double if we replace cowrie ESUs with the currently recognized species. This increase in error is the result of a simultaneous increase in the range of intraspecific variation and interspecific divergence, creating a wider overlap between the two in species than in ESUs. Intraspecific variability is substantially higher in traditional species than ESUs for all three metrics. The distribution of all intraspecific pairwise comparisons is multi-modal, reflecting the lumping of discrete ESUs (Figure 8A). The means of all intraspecific pairwise distances and theta are both three times as high within species (2.97%, θ = 1.86%) than within ESUs (0.81%, θ = 0.63%) (Figure 8). The range of interspecific divergence is also increased because numerous traditionally recognized cowrie species are not monophyletic in their COI, either because their coalescents have not sorted, or because they represent forms recognized by splitters that are not based on biological species. As a result, overlap between intra- and interspecific differences and error rates associated with thresholds both are greater when traditional species are used (Figure 9). The optimal threshold for recognized species is 5%, with an error rate of 33%. The 2.6% threshold, optimal for ESUs, yields a 37% error rate. Thus, thresholds fare poorly even in a thoroughly sampled phylogeny, if the basis for sampling is traditionally recognized species. This result is a strong warning against limited sampling to exemplars for taxa based on species checklists, even for relatively well-known groups. Had we not sampled the various subspecies of cowries and geographic locations as in the turbinids and limpets, we would have had a very different perspective on intra- and interspecific divergences.

#### Global versus regional sampling

This broad overlap contrasts with Hebert et al.'s [7] and Barrett and Hebert's [8] findings of a wide separation between intraspecific variation and interspecific divergence in a sample of North American birds and spiders. What causes this difference? This difference likely reflects differential intensity (number of samples per species/ESU) and scale (regional versus global) of sampling, rather than differences among birds, spiders, and snails. First, Hebert et al.'s [7] appraisal of intraspecific variation was limited, and thus they underestimated intraspecific variation [23,24]. Second, they substantially undersampled true sister species pairs [23,24,38], and thus overestimated interspecific divergence. Regional studies [1,7,8] undersample the most closely related species, which are frequently allopatric, and thus underestimate global error rates. The purported barcoding “gap” reported in these studies is the best-case scenario, and can only get worse (decrease or disappear) with increased intraspecific and interspecific sampling. Error rates are lower in regionally scaled analyses if the geographic scale of the study excludes allopatric sister taxa, thus artificially increasing observed interspecific divergence levels. For instance, a barcoding gap does exist if only cowries from the island of Moorea were investigated. The geographic scale where such reduction in error occurs is dependent on the geographic mode and scale of speciation of the group. While marine gastropods sampled at a single island would generally not include any allopatric sister taxa, terrestrial gastropods sampled at that same island may include many shallow sisters, if the landsnail group has undergone in situ radiation [39]. Consequently, error rates can be high even in geographically restricted analyses if diversification is local—through fine-scale allopatric speciation, sympatric speciation, polyploidy, or rapid attainment of sympatry following allopatry—or when invasive species homogenize the biota.

### Conclusions

Two principal elements are proposed in DNA barcoding: (1) the ability to assign an unknown sample to a known species, and (2) the ability to detect previously unsampled species as distinct. The prospect of assigning an unknown to a known is promising especially for well-known, comprehensively sampled groups that have been extensively studied by genetic and morphological taxonomy. In such globally comprehensive and well-circumscribed datasets, the majority of individuals (>96% in these snails) may be successfully identified by a short fragment of mtDNA. However, even in such extensively studied taxa, a certain percentage of young species (0%–8% in cowries) will not be discernable because of ancestral polymorphism. DNA barcoding is much less effective for identification in taxa where taxonomic scrutiny has not been thorough, and species recognition is limited to a few traditional character sets, untested by additional studies and tools. In such modestly known groups, which represent the bulk of life on Earth, many species will appear to be genetically non-monophyletic because of imperfect taxonomy [20], contributing to a high error rate for barcode-based identification. Thus, to create an effective environment for identification through barcoding, comprehensive, taxonomically thoroughly studied, comparative databases are necessary. The barcoding movement will play a leading role in generating the standards and protocols for establishing these databases, and facilitating their development.

The promise of barcoding for species discovery based on methodologies currently proposed should be tempered. The use of thresholds for species delineation is not promising and is strongly discouraged, as levels of overlap between intra- and interspecific differences are likely to be significant in most major clades, particularly within diverse yet poorly documented groups. Thresholds can be effective in screening for substantially divergent novel taxa, but our data indicate such use will overlook at least one-fifth of life's forms that are distinct but less divergent. More elegant methodologies will be required that incorporate principles of population genetics, knowledge of intraspecific variability, and sister group attributes. Identifications or discoveries may be placed within a statistical framework [40], allowing statements such as “based on the data at hand, sample X is 83% likely to be a member of taxon A.” The Data Analysis Working Group (DAWG) associated with the Consortium for the Barcoding of Life (CBOL) is pursuing these analytical challenges.

While the barcode is certainly a link out and can provide access to life's encyclopedia, this book needs to be written in collaboration with taxonomists, systematists, and ecologists, in an integrative taxonomic framework [17,41,42]. Barcoding on a global scale can only achieve high accuracy once the majority of evolutionary units have been sampled and taxonomically assessed. This critical first step was achieved for the studied gastropod taxa by centuries of careful, traditional taxonomic consideration (cowries) and large sample sizes (for all three). Without this initial phase, a threshold approach is likely to fail for ~20% of the taxa and individuals at the species discovery phase.

## Materials and Methods

We sequenced 2,026 cowries for 614 bp of COI mtDNA, the traditional Folmer primer region proposed for barcoding most metazoans. Two or more individuals were sequenced from 82% (216) of ESUs, ≥5 from 54% (143), and ≥10 from 23% (60). To maximize recovery of the greatest intraspecific variation and test for geographical structuring, sequences were generated from the most geographically distant populations available. Molecular methods followed standard procedures and are reviewed in Meyer [25,26], Kirkendale and Meyer [27], and Meyer et al. [28].

We used standard, tree-based methods to address accuracy of identification in a thoroughly sampled phylogeny using both a species-level and ESU approach. One exemplar from each recognized species (the nominal subspecies if the species included multiple subspecies) or each identified ESU was used as the reference “barcode” exemplar in topological comparisons. We randomly selected 1,000 sequences from the cowrie COI dataset, excluding barcode exemplars, and limiting representation of each species or ESU to 15 or ten sequences, respectively, to minimize bias toward well-sampled taxa. Hybrid individuals (see above) were excluded. These 1,000 sequences were tested one at a time, and their placement relative to the barcoding exemplars evaluated in both neighbor-joining (K2P) and parsimony phylogenies. Identification was considered correct if the sister taxon of the test sequence was the exemplar sequence of its corresponding species or ESU. Identification was considered incorrect if the sister taxon was wrong. If the random sequence fell below a node linking two recognized sister taxa including the corresponding species, the identification was considered ambiguous, as assignment to one or the other is equivocal, as the unknown could also represent a novel taxon. Similar analyses were performed with the turbinid (*n* = 200 from 278) and limpet (*n* = 100 from 125) datasets.

Pairwise K2P distances, theta, and coalescent depth were used to characterize intraspecific variation. Genetic distance between terminal taxa and their closest sister was used to characterize interspecific divergence. While the phylogenies used are based upon sequence data from two mtDNA markers (16S and COI: [26–28]), only COI was used for these analyses. The two most genetically distant individuals within each ESU (based on pairwise comparisons) were chosen to bookend genetic diversity and recover coalescent depth (maximum intra-ESU variability). These two individuals replaced the exemplar taxon used to construct the overall phylogeny (Figure 3). A likelihood ratio test (GTR + G with and without a clock enforced) was used to test for clock-like behavior (using only COI) in the resulting tree. A clock could not be falsified for turbinids and limpets (*p* > 0.05); but was falsified (*p* = 0.007) for cowries. Coalescent depths and interspecific divergence estimates throughout are based on topologies with a molecular clock enforced, although the overall cowrie data marginally rejected rate constancy. We estimated theta by calculating the average intraspecific difference using K2P distances. All analyses were conducted using PAUP* version 4.0b10 [43]. A listing of ESUs, number of individuals examined, interspecific divergence, and intraspecific metrics can be found in the supporting information for cowries (Table S1), turbinids (Table S2), and limpets (Table S3).

## Supporting Information

Figure S1 Barcoding Overlap in Turbinids (A) Relative distributions of intraspecific variability (coalescent depth—red) and interspecific divergence between ESUs (yellow). Note that the *x*-axis scale shifts to progressively greater increments above 0.01.
(B) Cumulative totals of false positives plus false negatives for each threshold value. The optimum threshold value is between 0.005 or 0.007 (1.0%–1.4%), where error is minimized at 7%.
(169 KB EPS).

Figure S2 Barcoding Gap in Limpets (A) Relative distributions of intraspecific variability (coalescent depth—red) and interspecific divergence between ESUs (yellow). Note that the *x*-axis scale shifts to progressively greater increments above 0.01.
(B) Cumulative totals of false positives plus false negatives for each threshold value. A gap exists at a threshold of 0.0085 (1.7%), where error is eliminated.
(166 KB EPS).

Table S1 ESU Listing for Cowries The table contains the taxon name, number of individuals sequenced, interspecific divergence (lineage) depth (GTR + G), coalescent depth (GTR + G), and estimated theta value (K2P). Asterisk (*) denotes direct developers, lacking planktonic larvae.
(343 KB DOC).

Table S2 ESU Listing for Turbinids The table contains the taxon name, number of individuals sequenced, interspecific divergence (lineage) depth (GTR + G), coalescent depth (GTR + G), and estimated theta value (K2P).
(55 KB DOC).

Table S3 ESU Listing for Limpets The table contains the taxon name, number of individuals sequenced, interspecific divergence (lineage) depth (GTR + G), coalescent depth (GTR + G), and estimated theta value (K2P).
(36 KB DOC).

### Accession Numbers

The GenBank (http://www.ncbi.nlm.nih.gov/Genbank) accession numbers for sequences discussed in this paper are: cowrie (AY161637–AY161846, AY534433–AY534503, and DQ206992–DQ207351), limpet (AY628240–AY628327), and turbinid (AY787233–AY787400). The complete datasets are available also by request from CPM or at the Cowrie Genetic Database Web site (http://www.flmnh.ufl.edu/cowries).

## Abbreviations

COI: cytochrome *c* oxidase I
ESU: evolutionary significant unit
mtDNA: mitochondrial DNA
NUMT: nuclear copy of mtDNA
