# Supplementary material for: DNA Barcoding: Error Rates Based on Comprehensive Sampling
Source: PLoS Biol. 2005 Nov 29;3(12):e422. doi: 10.1371/journal.pbio.0030422 (PMC1287506; doi:10.1371/journal.pbio.0030422)
Supplement: Supplementary file 1 [file pbio.0030422.st001.doc]

Table S1. ESU listing for cowries. The table contains the taxon name, number of individuals sequenced, interspecific divergence (lineage) depth (GTR+G), coalescent depth (GTR+G), and estimated theta value (K2P). Asterisk (*) denotes direct developers, lacking planktonic larvae.

* denotes direct developer, lacking planktonic larvae

| **Taxon** | **# indiv** | **lineage** | **coalescent** | **Theta**  **(N ≥ 10)** |
| --- | --- | --- | --- | --- |
| Nesiocypraea teramachii | 2 | 0.25365 | 0.00236 |  |
| Ipsa childreni | 2 | 0.25365 | 0.00634 |  |
| Propustularia surinamensis | 1 | 0.30983 |  |  |
| Monetaria annulus | 80 | 0.11674 | 0.00752 | 0.00758 |
| Monetaria moneta | 10 | 0.10537 | 0.00447 | 0.00397 |
| Monetaria caputserpentis caputserpentis | 15 | 0.01388 | 0.00561 | 0.00743 |
| Monetaria caputdraconis | 8 | 0.01388 | 0.00395 |  |
| Monetaria caputserpentis caputophidii | 10 | 0.01388 | 0.00535 | 0.00776 |
| Perisserosa guttata | 2 | 0.14925 | 0 |  |
| Erosaria irrorata | 2 | 0.08917 | 0.00222 |  |
| Erosaria albuginosa | 2 | 0.03229 | 0.0027 |  |
| Erosaria poraria | 5 | 0.03229 | 0.00533 |  |
| Erosaria spurca | 6 | 0.00895 | 0.00696 |  |
| Erosaria acicularis | 5 | 0.00895 | 0.00607 |  |
| Erosaria cernica | 7 | 0.0277 | 0.00502 |  |
| Erosaria bernardi kingae | 1 | 0.01011 |  |  |
| Erosaria thomasi | 5 | 0.0101 | 0.00135 |  |
| Erosaria englerti | 6 | 0.05059 | 0.00525 |  |
| Erosaria beckii | 5 | 0.00488 | 0.0046 |  |
| Erosaria macandrewi | 1 | 0.00488 |  |  |
| Erosaria ocellata | 5 | 0.01969 | 0.003 |  |
| Erosaria gangranosa | 2 | 0.01969 | 0.00232 |  |
| Erosaria boivinii | 5 | 0.02449 | 0.00082 |  |
| Erosaria labrolineata | 5 | 0.03655 | 0.00139 |  |
| Erosaria miliaris | 5 | 0.06278 | 0.00775 |  |
| Erosaria lamarckii | 10 | 0.06278 | 0.01367 | 0.01204 |
| Erosaria nebrites | 5 | 0.05273 | 0.00155 |  |
| Erosaria erosa | 6 | 0.02138 | 0.00269 |  |
| Erosaria erosa cf. chlorizans | 10 | 0.02138 | 0.00395 | 0.00447 |
| Erosaria turdus | 6 | 0.1114 | 0.0035 |  |
| Erosaria marginalis | 2 | 0.04794 | 0.00924 |  |
| Erosaria citrina | 2 | 0.04794 | 0.0107 |  |
| Erosaria helvola helvola | 91 | 0.08917 | 0.0131 | 0.01247 |
| Cryptocypraea dillwyni | 2 | 0.14894 | 0 |  |
| Staphylaea limacina limacina | 5 | 0.01696 | 0.00324 |  |
| Staphylaea limacina interstincta | 5 | 0.01697 | 0.00559 |  |
| Staphylaea staphylaea laevigata | 2 | 0.04216 | 0.00633 |  |
| Staphylaea staphylaea staphylaea | 10 | 0.03933 | 0.00244 | 0.00137 |
| Staphylaea semiplota | 1 | 0.03932 |  |  |
| Nucleolaria nulceus | 13 | 0.13827 | 0.00839 | 0.01018 |
| Trona stercoraria | 5 | 0.19234 | 0.00081 |  |
| Annepona mariae | 5 | 0.19234 | 0.0052 |  |
| Chelycypraea testudinaria | 5 | 0.19736 | 0.00254 |  |
| Austrocypraea reevei* | 2 | 0.1653 | 0.00514 |  |
| Arestoides argus argus | 2 | 0.022 | 0.007 |  |
| Arestoides argus contrastriata | 1 | 0.022 |  |  |
| Lyncina nivosa | 2 | 0.08764 | 0.00261 |  |
| Lyncina broderipii | 1 | 0.08765 |  |  |
| Lyncina leucodon | 2 | 0.06913 | 0.00239 |  |
| Lyncina aurantium | 2 | 0.06913 | 0.00076 |  |
| Lyncina porteri | 1 | 0.16043 |  |  |
| Lyncina sulcidentata | 2 | 0.00487 | 0.00455 |  |
| Lyncina kuroharai | 1 | 0.00487 |  |  |
| Lyncina schilderorum | 2 | 0.07766 | 0.00721 |  |
| Lyncina leviathan | 7 | 0.04972 | 0.00585 |  |
| Lyncina carneola carneola | 21 | 0.04972 | 0.01412 | 0.01323 |
| Lyncina ventriculus | 6 | 0.01071 | 0.00209 |  |
| Lyncina ventriculus (Xmas IO) | 1 | 0.01071 |  |  |
| Lyncina camelopardalis | 3 | 0.1389 | 0.00153 |  |
| Lyncina lynx | 5 | 0.12428 | 0.00754 |  |
| Lyncina vitellus | 6 | 0.12427 | 0.00669 |  |
| Luria isabella | 20 | 0.0784 | 0.00777 | 0.00980 |
| Luria tessellata | 5 | 0.07839 | 0.00703 |  |
| Luria cinerea | 6 | 0.11024 | 0.00547 |  |
| Luria lurida | 6 | 0.11024 | 0.00611 |  |
| Luria pulchra | 3 | 0.11024 | 0.00596 |  |
| Talparia talpa | 3 | 0.17734 | 0.01145 |  |
| Pustularia bistrinotata bistrinotata | 5 | 0.00955 | 0.00546 |  |
| Pustularia bistrinotata sublaevis | 5 | 0.00955 | 0.00794 |  |
| Pustularia bistrinotata keelingensis | 1 | 0.02101 |  |  |
| Pustularia mauiensis | 6 | 0.0396 | 0.0033 |  |
| Pustularia cicercula | 11 | 0.06693 | 0.00934 | 0.00991 |
| Pustularia margarita | 5 | 0.06693 | 0.01064 |  |
| Pustularia globulus globulus | 5 | 0.01235 | 0.00577 |  |
| Pustularia globulus brevirostris | 2 | 0.01235 | 0.0079 |  |
| Neobernaya spadicea | 5 | 0.16477 | 0.00169 |  |
| Pseudozonaria annettae | 2 | 0.12811 | 0.00236 |  |
| Pseudozonaria arabicula | 2 | 0.06169 | 0.01116 |  |
| Pseudozonaria nigropunctata | 1 | 0.06169 |  |  |
| Pseudozonaria robertsi | 5 | 0.07087 | 0.00494 |  |
| Schilderia achatidea | 1 | 0.12173 |  |  |
| Zonaria pyrum | 3 | 0.0789 | 0.00659 |  |
| Zonaria sanguinolenta | 2 | 0.07891 | 0 |  |
| Zonaria picta | 2 | 0.07891 | 0.0044 |  |
| Zonaria zonaria | 5 | 0.08622 | 0.00416 |  |
| Notocypraea comptoni* | 17 | 0.07171 | 0.01091 | 0.00737 |
| Notocypraea angustata (P. Lincoln)* | 2 | 0.0137 | 0 |  |
| Notocypraea angustata* | 5 | 0.0137 | 0.00343 |  |
| Notocypraea declivis* | 6 | 0.06033 | 0.0031 |  |
| Notocypraea piperita* | 13 | 0.06639 | 0.00476 | 0.00463 |
| Notocypraea pulicaria* | 1 | 0.06638 |  |  |
| Notocypraea hartsmithi* | 1 | 0.04867 |  |  |
| Notocypraea cf. vercornis* | 1 | 0.04867 |  |  |
| Cypraeovula castanea* | 2 | 0.00179 | 0 |  |
| Cypraeovula iutsui* | 2 | 0.00179 | 0 |  |
| Cypraeovula connelli* | 1 | 0.0167 |  |  |
| Cypraeovula fuscorubra* | 2 | 0.02564 | 0.0074 |  |
| Cypraeovula capensis* | 3 | 0.0038 | 0.00237 |  |
| Cypraeovula coronata* | 2 | 0.01439 | 0.0032 |  |
| Cypraeovula edentula* | 4 | 0.00623 | 0.00263 |  |
| Cypraeovula algoensis* | 3 | 0.00623 | 0.00403 |  |
| Bistolida owenii vasta | 1 | 0.05302 |  |  |
| Bistolida owenii owenii | 2 | 0.05303 | 0.00837 |  |
| Bistolida erythraeensis | 2 | 0.08844 | 0.00358 |  |
| Bistolida goodallii fuscomaculata | 3 | 0.01402 | 0.00263 |  |
| Bistolida goodallii goodallii | 1 | 0.01402 |  |  |
| Bistolida hirundo | 13 | 0.099 | 0.01008 | 0.00997 |
| Bistolida ursellus | 2 | 0.0435 | 0.00513 |  |
| Bistolida ursellus (Andaman) | 1 | 0.0435 |  |  |
| Bistolida kieneri depriesteri | 4 | 0.02665 | 0.00323 |  |
| Bistolida kieneri schneideri | 7 | 0.02665 | 0.01117 |  |
| Bistolida kieneri kieneri | 5 | 0.04755 | 0.00485 |  |
| Bistolida stolida stolida | 12 | 0.03958 | 0.00712 | 0.00890 |
| Bistolida stolida clavicola | 8 | 0.01791 | 0.00439 |  |
| Bistolida diagues | 2 | 0.00796 | 0.002 |  |
| Bistolida stolida rubiginosa | 1 | 0.00796 |  |  |
| Palmadusta asellus bitaeniata | 2 | 0.06219 | 0.00288 |  |
| Palmadusta asellus vespacea | 5 | 0.01964 | 0.0073 |  |
| Palmadusta asellus asellus | 2 | 0.01964 | 0.00093 |  |
| Palmadusta artuffeli | 8 | 0.01448 | 0.00766 |  |
| Palmadusta clandestina cf. candida | 16 | 0.01448 | 0.00999 | 0.01056 |
| Palmadusta clandestina clandestina | 5 | 0.01442 | 0.00466 |  |
| Palmadusta clandestina passerina | 6 | 0.01443 | 0.00784 |  |
| Palmadusta diluculum | 2 | 0.07008 | 0.01042 |  |
| Palmadusta lentiginosa | 5 | 0.10057 | 0.0026 |  |
| Palmadusta ziczac ziczac | 2 | 0.03759 | 0 |  |
| Palmadusta ziczac misella | 2 | 0.03759 | 0.00082 |  |
| Palmadusta lutea | 6 | 0.12102 | 0.00389 |  |
| Palmadusta saulae | 3 | 0.12102 | 0.00158 |  |
| Palmadusta contaminata contaminata | 5 | 0.01474 | 0.00563 |  |
| Palmadusta contaminata distans | 1 | 0.01473 |  |  |
| Ovatipsa chinensis amiges | 10 | 0.01225 | 0.0038 | 0.00313 |
| Ovatipsa chinensis chinensis | 7 | 0.01225 | 0.00553 |  |
| Ovatipsa coloba | 7 | 0.10528 | 0.01024 |  |
| Talostolida rashleighana rashleighana | 1 | 0.04833 |  |  |
| Talostolida rashleighana pseudoteres | 1 | 0.04833 |  |  |
| Talostolida teres | 11 | 0.09093 | 0.01149 | 0.00741 |
| Talostolida pellucens | 27 | 0.0686 | 0.00684 | 0.00556 |
| Talostolida subteres | 6 | 0.05481 | 0.00078 |  |
| Talostolida latior | 1 | 0.05481 |  |  |
| Cribrarula cribraria cribraria | 31 | 0.00607 | 0.00432 | 0.00337 |
| Cribrarula "exmouthensis" abrolhensis | 5 | 0.00607 | 0.00206 |  |
| Cribrarula cribraria melwardi | 8 | 0.00678 | 0.00074 |  |
| Cribrarula "exmouthensis" rottnestensis | 4 | 0.01856 | 0.00081 |  |
| Cribrarula exmouthensis exmouthensis | 1 | 0.00859 |  |  |
| Cribrarula exmouthensis magnifica | 1 | 0.00859 |  |  |
| Cribrarula cribraria australiensis | 7 | 0.01028 | 0.0046 |  |
| Cribrarula cribraria ganteri | 1 | 0.01029 |  |  |
| Cribrarula cribraria cf. abaliena | 1 | 0.01029 |  |  |
| Cribrarula cribraria n spp. 1 (Sey) | 2 | 0.02288 | 0 |  |
| Cribrarula cribraria gaspardi | 2 | 0.0387 | 0.00234 |  |
| Cribrarula fallax | 2 | 0.0431 | 0 |  |
| Cribrarula pellisserpentis | 3 | 0.03022 | 0.00226 |  |
| Cribrarula cribraria comma | 7 | 0.00935 | 0.00361 |  |
| Cribrarula esontropia francescoi | 2 | 0.00437 | 0.00083 |  |
| Cribrarula esontropia esontropia | 4 | 0.00438 | 0.00194 |  |
| Cribrarula cumingii cumingii | 12 | 0.01728 | 0.00707 | 0.00524 |
| Cribrarula cumingii astaryi | 10 | 0.01728 | 0.00536 | 0.00436 |
| Cribrarula gaskoini | 11 | 0.06324 | 0.00829 | 0.00822 |
| Cribrarula catholicorum | 4 | 0.06325 | 0.00574 |  |
| Austrasiatica hirasei | 1 | 0.06616 |  |  |
| Austrasiatica langfordi | 2 | 0.06616 | 0.00297 |  |
| Austrasiatica sakurai | 1 | 0.07179 |  |  |
| Palmulacypraea omii | 2 | 0.00726 | 0.00088 |  |
| Palmulacypraea katsuae | 1 | 0.00725 |  |  |
| Palmulacypraea musumea | 1 | 0.05747 |  |  |
| Purpuradusta microdon microdon | 5 | 0.00983 | 0.00238 |  |
| Purpuradusta microdon chrysalis | 1 | 0.00982 |  |  |
| Purpuradusta barbieri | 1 | 0.08593 |  |  |
| Purpuradusta hammondae | 5 | 0.09945 | 0.00868 |  |
| Purpuradusta minoridens | 7 | 0.06205 | 0.01771 |  |
| Purpuradusta oryzaeformis | 5 | 0.06205 | 0.00454 |  |
| Purpuradusta serrulifera | 4 | 0.12435 | 0.00861 |  |
| Purpuradusta gracilis gracilis | 12 | 0.0412 | 0.00813 | 0.00751 |
| Purpuradusta gracilis notata | 4 | 0.04119 | 0.0009 |  |
| Purpuradusta fimbriata fimbriata | 10 | 0.02771 | 0.00874 | 0.00661 |
| Purpuradusta fimbriata unifasciata | 51 | 0.02771 | 0.01812 | 0.01987 |
| Melicerona listeri melvilli | 5 | 0.04255 | 0.00896 |  |
| Melicerona listeri listeri | 5 | 0.01013 | 0.00585 |  |
| Melicerona felina | 5 | 0.01013 | 0.00513 |  |
| Blasicrura pallidula pallidula | 2 | 0.03814 | 0 |  |
| Blasicrura pallidula rhinoceros | 3 | 0.01938 | 0.00254 |  |
| Blasicrura pallidula cf. vivia | 1 | 0.01674 |  |  |
| Blasicrura summersi | 2 | 0.01674 | 0 |  |
| Blasicrura interrupta | 4 | 0.05134 | 0.00593 |  |
| Eclogavena luchuana | 2 | 0.02591 | 0 |  |
| Eclogavena dayritiana | 5 | 0.02591 | 0 |  |
| Eclogavena coxeni | 2 | 0.01123 | 0.00067 |  |
| Eclogavena quadrimaculata quadrimaculata | 7 | 0.01122 | 0.00391 |  |
| Eclogavena quadrimaculata thielei | 1 | 0.01937 |  |  |
| Notadusta punctata berinii | 9 | 0.06239 | 0.01786 |  |
| Notadusta punctata (Andaman) | 5 | 0.0584 | 0.00223 |  |
| Notadusta punctata trizonata | 9 | 0.01799 | 0.00385 |  |
| Notadusta punctata punctata | 20 | 0.018 | 0.00814 | 0.00518 |
| Notadusta martini | 3 | 0.09894 | 0.00373 |  |
| Notadusta hungerfordi | 1 | 0.12125 |  |  |
| Contradusta walkeri | 6 | 0.0254 | 0.01368 |  |
| Contradusta bregeriana | 3 | 0.0254 | 0.00498 |  |
| Contradusta barclayi | 1 | 0.08952 |  |  |
| Contradusta pulchella | 2 | 0.08952 | 0.0015 |  |
| Erronea xanthodon | 2 | 0.11398 | 0.00103 |  |
| Erronea pallida | 1 | 0.10969 |  |  |
| Erronea vredenburgi | 5 | 0.07823 | 0.00812 |  |
| Erronea rabaulensis | 1 | 0.07823 |  |  |
| Erronea subviridis dorsalis | 6 | 0.02897 | 0.00673 |  |
| Erronea subviridis subviridis | 3 | 0.02897 | 0.00879 |  |
| Erronea adusta | 5 | 0.03479 | 0.00243 |  |
| Erronea onyx | 11 | 0.03478 | 0.00847 | 0.00972 |
| Erronea ovum chrysostoma | 13 | 0.02746 | 0.00237 | 0.00147 |
| Erronea caurica caurica | 22 | 0.02511 | 0.01251 | 0.01009 |
| Erronea errones | 30 | 0.02511 | 0.00925 | 0.00918 |
| Erronea caurica cf. derosa | 14 | 0.03064 | 0.00677 | 0.00510 |
| Erronea caurica spp. 1 | 24 | 0.03064 | 0.00611 | 0.00497 |
| Erronea caurica dracaena | 12 | 0.0047076 | 0.0047 | 0.00275 |
| Erronea caurica elongata | 8 | 0.00473 | 0.00074 |  |
| Erronea caurica quinquefasciata | 17 | 0.00926 | 0.00719 | 0.00504 |
| Erronea ovum ovum | 15 | 0.01223 | 0.00564 | 0.00571 |
| Erronea ovum palauensis | 7 | 0.01223 | 0.00066 |  |
| Erronea cylindrica lenella | 8 | 0.01373 | 0 |  |
| Erronea cylindrica cylindrica | 10 | 0.01373 | 0.00899 | 0.00422 |
| Erronea pyriformis | 1 | 0.07722 |  |  |
| Barycypraea teulerei* | 1 | 0.10758 |  |  |
| Barycypraea fultoni* | 4 | 0.10758 | 0.00361 |  |
| Zoila marginata ketyana* | 30 | 0.00706 | 0.00436 | 0.00373 |
| Zoila marginata marginata* | 13 | 0.00705 | 0.00304 | 0.00260 |
| Zoila venusta* | 23 | 0.06208 | 0.00171 | 0.00095 |
| Zoila rosselli* | 16 | 0.05681 | 0.00501 | 0.00281 |
| Zoila decipiens* | 6 | 0.00519 | 0.00128 |  |
| Zoila mariellae* | 1 | 0.00519 |  |  |
| Zoila perlae/eludens* | 20 | 0.01301 | 0.00124 | 0.00033 |
| Zoila friendii* | 92 | 0.01301 | 0.00711 | 0.00609 |
| Mauritia arabica arabica | 69 | 0.0507 | 0.00767 | 0.00494 |
| Mauritia arabica asiatica | 52 | 0.03892 | 0.00473 | 0.00339 |
| Mauritia arabica immanis | 19 | 0.03893 | 0.00642 | 0.00541 |
| Mauritia maculifera maculifera | 21 | 0.00718 | 0.0033 | 0.00175 |
| Mauritia maculifera scindata | 19 | 0.00718 | 0.00297 | 0.00226 |
| Mauritia maculifera martybealsi | 10 | 0.01889 | 0.00254 | 0.00247 |
| Mauritia eglantina | 6 | 0.10468 | 0.00531 |  |
| Mauritia histrio | 6 | 0.10467 | 0.00562 |  |
| Mauritia grayana | 13 | 0.11816 | 0.00604 | 0.00484 |
| Mauritia mauritiana | 6 | 0.10785 | 0.01182 |  |
| Mauritia depressa dispersa | 11 | 0.00628 | 0.00474 | 0.00753 |
| Mauritia depressa depressa | 11 | 0.00629 | 0.00489 | 0.00664 |
| Mauritia scurra indica | 7 | 0.0822 | 0.0068 |  |
| Mauritia scurra scurra | 5 | 0.0822 | 0.0008 |  |
| Leporicypraea mappa mappa | 15 | 0.12086 | 0.00614 | 0.00431 |
| Leporicypraea mappa aliwalensis | 1 | 0.0387 |  |  |
| Leporicypraea mappa rosea | 1 | 0.0387 |  |  |
| Leporicypraea geographica | 18 | 0.06691 | 0.0151 | 0.01494 |
| Leporicypraea mappa viridis | 17 | 0.02585 | 0.01172 | 0.00780 |
| Leporicypraea mappa admirabilis | 6 | 0.02585 | 0.01868 |  |
| Leporicypraea valentia | 3 | 0.1692 | 0.00077 |  |
| Macrocypraea cervinetta | 5 | 0.08948 | 0.01016 |  |
| Macrocypraea zebra | 3 | 0.04486 | 0.00328 |  |
| Macrocypraea cervus | 8 | 0.04486 | 0.00916 |  |
| Cypraea tigris (IO) | 23 | 0.08987 | 0.01026 | 0.00896 |
| Cypraea tigris (PO) | 54 | 0.0781 | 0.01245 | 0.01189 |
| Cypraea pantherina | 4 | 0.0781 | 0.00165 |  |
| Muracypraea mus* | 1 | 0.21984 |  |  |
| Umbilia hesitata* | 11 | 0.03103 | 0 | 0.00000 |
| Umbilia capricornica* | 5 | 0.00896 | 0.00137 |  |
| Umbilia petilirostris* | 1 | 0.00896 |  |  |
| Umbilia armeniaca* | 10 | 0.05881 | 0.00086 | 0.00078 |
