# Supplementary material for: DNA Barcoding: Error Rates Based on Comprehensive Sampling
Source: PLoS Biol. 2005 Nov 29;3(12):e422. doi: 10.1371/journal.pbio.0030422 (PMC1287506; doi:10.1371/journal.pbio.0030422)
Supplement: Supplementary file 2 [file pbio.0030422.st002.doc]

Table S2. ESU listing for turbinids. The table contains the taxon name, number of individuals sequenced, interspecific divergence (lineage) depth (GTR+G), coalescent depth (GTR+G), and estimated theta value (K2P).

Table S2. Turbinid Data (*Astralium rhodostomum* complex)

| **Taxon** | **# indiv** | **lineage** | **coalescent** | **Theta**  **(N > 5)** |
| --- | --- | --- | --- | --- |
| Marianas B | 18 | 0.02748 | 0.00698 | 0.00643 |
| Tonga, Samoa, Niue A | 11 | 0.02101 | 0.0052 | 0.00404 |
| Fiji A | 9 | 0.02101 | 0.0049 | 0.00680 |
| New Caledonia B | 6 | 0.04236 | 0.00481 | 0.00626 |
| Japan B | 2 | 0.11173 | 0.0031 |  |
| New Britain B | 14 | 0.00914 | 0.00279 | 0.00152 |
| Rapa A | 9 | 0.00756 | 0.00274 | 0.00220 |
| Marshalls B | 12 | 0.03964 | 0.00248 | 0.00368 |
| New Caledonia A | 11 | 0.02477 | 0.00239 | 0.00206 |
| Ryukyu B | 15 | 0.09323 | 0.00234 | 0.00192 |
| Palau B | 18 | 0.00902 | 0.00213 | 0.00643 |
| Tuamotu B | 10 | 0.03274 | 0.0021 | 0.00292 |
| Kosrae B | 3 | 0.03699 | 0.00184 |  |
| Central Visayas B | 11 | 0.00505 | 0.00181 | 0.00174 |
| Marianas A | 2 | 0.02101 | 0.00159 | 0.00372 |
| Cooks A | 13 | 0.00756 | 0.00152 | 0.00132 |
| Fiji B | 9 | 0.00643 | 0.00144 | 0.00115 |
| Ryukyu A | 9 | 0.02477 | 0.00137 | 0.00153 |
| Yap B | 24 | 0.00901 | 0.00137 | 0.00146 |
| Palawan B | 3 | 0.00506 | 0.00086 |  |
| PNG / QLD B | 13 | 0.03914 | 0.00076 | 0.00024 |
| Cocos Keeling B | 4 | 0.00776 | 0.00074 |  |
| Tonga, Samoa, Niue B | 16 | 0.00643 | 0.00074 | 0.00100 |
| Manus B | 9 | 0.00915 | 0.00073 | 0.00105 |
| Societies B | 8 | 0.03273 | 0.00073 | 0.00429 |
| Christmas Is. (IO) B | 4 | 0.00776 | 0.00072 |  |
| Gulf of Thailand B | 6 | 0.1557 | 0.0007 | 0.00107 |
| Andaman Sea B | 1 | 0.03148 | 0 |  |
| Sulawesi B | 1 | 0.01714 | 0 |  |
| Line B | 2 | 0.11173 | 0 |  |
