# Supplementary material for: DNA Barcoding: Error Rates Based on Comprehensive Sampling
Source: PLoS Biol. 2005 Nov 29;3(12):e422. doi: 10.1371/journal.pbio.0030422 (PMC1287506; doi:10.1371/journal.pbio.0030422)
Supplement: Supplementary file 3 [file pbio.0030422.st003.doc]

Table S3. ESU listing for limpets. The table contains the taxon name, number of individuals sequenced, interspecific divergence (lineage) depth (GTR+G), coalescent depth (GTR+G), and estimated theta value (K2P).

Table S3. Limpet Data (*Patelloida profunda* group)

| **Taxon** | **# indiv** | **lineage** | **coalescent** | **Theta**  **(N > 3)** |
| --- | --- | --- | --- | --- |
| Sulawesi | 10 | 0.00885 | 0.00202 | 0.0024 |
| Bali / Philippines | 20 | 0.00885 | 0.00337 | 0.0016 |
| Palau | 11 | 0.01381 | 0.00299 | 0.0009 |
| Zanzibar | 14 | 0.02633 | 0.00246 | 0.0016 |
| Mauritius (P. mauritiana) | 4 | 0.02633 | 0.00513 | 0.0063 |
| South Africa (P. albonotata) | 10 | 0.03951 | 0 | 0 |
| Marianas (P. chammarorum) | 19 | 0.04518 | 0.00163 | 0.0007 |
| Oman (P. p. omanensis) | 11 | 0.06632 | 0.00319 | 0.0015 |
| South Java | 4 | 0.08483 | 0 | 0 |
| Cooks (P. conoidalis) | 13 | 0.08483 | 0.00286 | 0.0024 |
| Reunion (P. profunda) | 5 | 0.73207 | 0.00421 | 0.0034 |
| Caribbean (P. pustulata) | 4 | 0.73208 | 0.00837 | 0.0086 |
